# Extracting the central crop row with CCRDNet for universal in-row navigation in agriculture

**DOI:** 10.3389/fpls.2026.1744637

**Published:** 2026-02-02

**Authors:** Hao Zheng, Qiang Wang

**Affiliations:** 1 Department of Control Science and Engineering, Harbin Institute of Technology, Harbin, Heilongjiang, China; 2 National Key Laboratory of Smart Farm Technologies and Systems, Harbin Institute of Technology, Harbin, Heilongjiang, China

**Keywords:** central crop row detection, image processing, machine vision, navigation line extraction, zero-shot

## Abstract

Deep learning has recently shown strong potential in crop row detection for navigation line extraction. However, existing approaches often rely on dataset-specific customization and extensive image preprocessing, limiting their practicality in real-world agricultural scenarios. In contrast, human operators can instinctively navigate machinery by simply following the central crop row. Inspired by this observation, we propose a novel strategy that directly extracts the central crop row as the navigation line. To support this paradigm, we introduce a three-class annotation scheme—background, vegetation, and central crop row—where the vegetation class serves as an auxiliary supervisory signal to provide structural constraints and guide accurate localization. A consistent annotation width of crop row is applied across all samples to enable the model to learn invariant structural features. We develop CCRDNet (Central Crop Row Detection Network), which predicts the central row position and subsequently fits the navigation line using the least-squares method. A dataset of 7,367 images comprising eight crop types across diverse environments was collected, yet only 400 images—from two crop types in eight environments—were used for training. Despite the limited supervision, the proposed method achieved a navigation line extraction accuracy of 95.57% with an average angle error of 1.13°. CCRDNet is lightweight, requiring only 0.033M parameters, and operates at 86.76 FPS on an RTX 3060 GPU and 48.78 FPS on a Jetson Orin NX. These results demonstrate that the proposed approach not only simplifies the navigation pipeline but also enables zero-shot generalization across previously unseen environments, fully satisfying the real-time requirements of agricultural machinery.

## 1 Introduction

With the rapid advancement of artificial intelligence, intelligent agricultural machinery has become increasingly prevalent in modern farming. Currently, two major navigation technologies are employed in field operations: satellite-based positioning systems and vision-based navigation systems. Compared with satellite positioning, vision-based navigation provides higher accuracy and reliability by directly perceiving crop distribution and environmental variations in real time. Among all field features, crop rows serve as the most essential visual cues, making their extraction a critical step in vision-based navigation.

Traditional approaches typically begin by segmenting vegetation based on color differences between crops and background soil or weeds. Morphological operations were then applied to suppress noise (García-Santillán et al., 2017, 2018), while other studies removed irrelevant regions according to connected component size (Gong et al., 2021; Rabab et al., 2021). To identify crop row feature points, Ma et al. (2022) divided images into horizontal strips and used vertical projections to locate crop positions, selecting the central pixel of each strip as the feature point. When multiple crop rows were present, techniques such as region search (He et al., 2021) or improved K-means clustering (Zhang et al., 2022) were adopted to group feature points. For line fitting, least-squares regression (Yang et al., 2022a), Hough transform (Winterhalter et al., 2018), and adaptive RANSAC methods (Xie et al., 2021b) were widely utilized. Some studies further introduced vanishing point estimation to enhance crop row localization (Li et al., 2023a).

Beyond explicit crop extraction, several researchers explored the central inter-row path as a navigation reference. Zhou et al. (2021) defined a region of interest enclosing two adjacent central rows, while Wang et al. (2021) and Chen et al. (2020) directly extracted the middle path and fitted the navigation line based on its feature points.

In recent years, deep neural networks (DNNs) have achieved remarkable success in object detection and image segmentation, motivating their adoption in agricultural perception tasks. Detection models such as the YOLO series (Diao et al., 2023b; Ruan et al., 2023; Wang et al., 2023) have demonstrated strong robustness against illumination variation, weed interference, and occlusion. A pioneering shift was introduced by Adhikari et al. (2019, 2020), who replaced pixel-level segmentation with semantic representations of row structures, enabling the simultaneous prediction of all crop rows. Subsequent studies (Bah et al., 2020; Cao et al., 2022; Diao et al., 2023a; Yang et al., 2022b) validated the feasibility of deep learning–based crop row extraction on multi-domain datasets, while Chen et al. (2021) directly inferred the central inter-row path. Other works employed detection-based region-of-interest (ROI) extraction (Yang et al., 2023) or end-to-end line prediction frameworks (Li et al., 2023b).

Despite these advances, most existing approaches still follow a multi-stage pipeline comprising: object (crop, crop row, or road) extraction, feature points extraction (via vertical projections), feature points grouping (e.g., K-means clustering), and line fitting. Deep learning only improves object extraction robustness, and these methods remain highly dependent on dataset specificity and tend to generalize poorly across crop species, growth stages, or environments. In many cases, a model trained on one crop species—or even a specific growth stage—fails to generalize to others. Consequently, when deployed in real fields, each change in crop variety or phenological stage often requires collecting new data, rebuilding the dataset, re-tuning parameters, or even retraining the model, which may miss the optimal time window for actual field operation.

Moreover, the subsequent steps still rely heavily on handcrafted algorithms and manually tuned parameters. Such pipelines, especially in feature points extraction and grouping, are highly sensitive to the varying number of visible crop rows within the camera’s field of view, which may fluctuate due to turns, terrain undulations, or row curvature. A changing number of candidate rows can cause incorrect clustering or region assignments, leading to navigation drift. Small inconsistencies in network outputs—such as detection gaps or local discontinuities—are often amplified in downstream steps, resulting in line-fitting failures or unstable navigation control. Environmental disturbances, including dense weeds, shadow interference, soil reflectance, and lodged crops, further exacerbate instability, often requiring frequent manual recalibration. These limitations highlight the need for a more streamlined and inherently robust framework that minimizes reliance on fragile intermediate steps.

In contrast, human operators do not explicitly extract every crop row or feature point. Instead, they intuitively align machinery by visually following the central crop row, regardless of crop species, morphology, or growth stage. This observation suggests an implicit yet powerful prior: the central crop row serves as a universal and stable navigation reference across diverse agricultural settings.

Motivated by this observation, we propose a streamlined paradigm that focuses solely on detecting the central crop row as the navigation line. Specifically, a semantic segmentation network outputs three classes: background, vegetation, and the central crop row. The navigation line is then derived by fitting the largest connected component in the central row class—without requiring feature extraction, clustering, or multi-row association. This design fundamentally differs from traditional multi-row extraction pipelines in several key aspects: (1) single-row focus eliminates error propagation across multiple detection stages; (2) direct supervision on the navigation target removes the need for auxiliary features and post-processing; and (3) robustness to structural changes is achieved, as the model handles varying numbers of visible rows without algorithmic reconfiguration. The overall pipeline is significantly simplified, and generalization capability is markedly enhanced. Consequently, navigation reliability depends on learned visual features rather than handcrafted rules, ensuring superior stability, transferability, and deployment efficiency. The main contributions of this work are summarized as follows:

- A novel navigation paradigm is proposed that directly detects the central crop row as the navigation line in a human-like manner. This strategy simplifies the navigation process, demonstrates robust performance in zero-shot environments that were never encountered during training, and requires only a small amount of training data.
- A large-scale, diverse dataset is collected, containing 7,367 images from eight crop species across multiple growth stages and environmental conditions. The dataset is made publicly available^1^to facilitate future research in agricultural navigation.
- A novel annotation strategy is introduced by incorporating a vegetation class, which serves as an auxiliary supervisory signal to provide structural constraints and guide the localization of the central row. The consistency of annotation width enables the model to learn the core structural characteristics of crop rows more effectively.
- A lightweight neural network architecture, CCRDNet, is developed, which achieves high accuracy and real-time performance with only 0.033M parameters.

## 2 Materials and methods

### 2.1 Novel scheme of navigation line extraction

**Figure 1:**
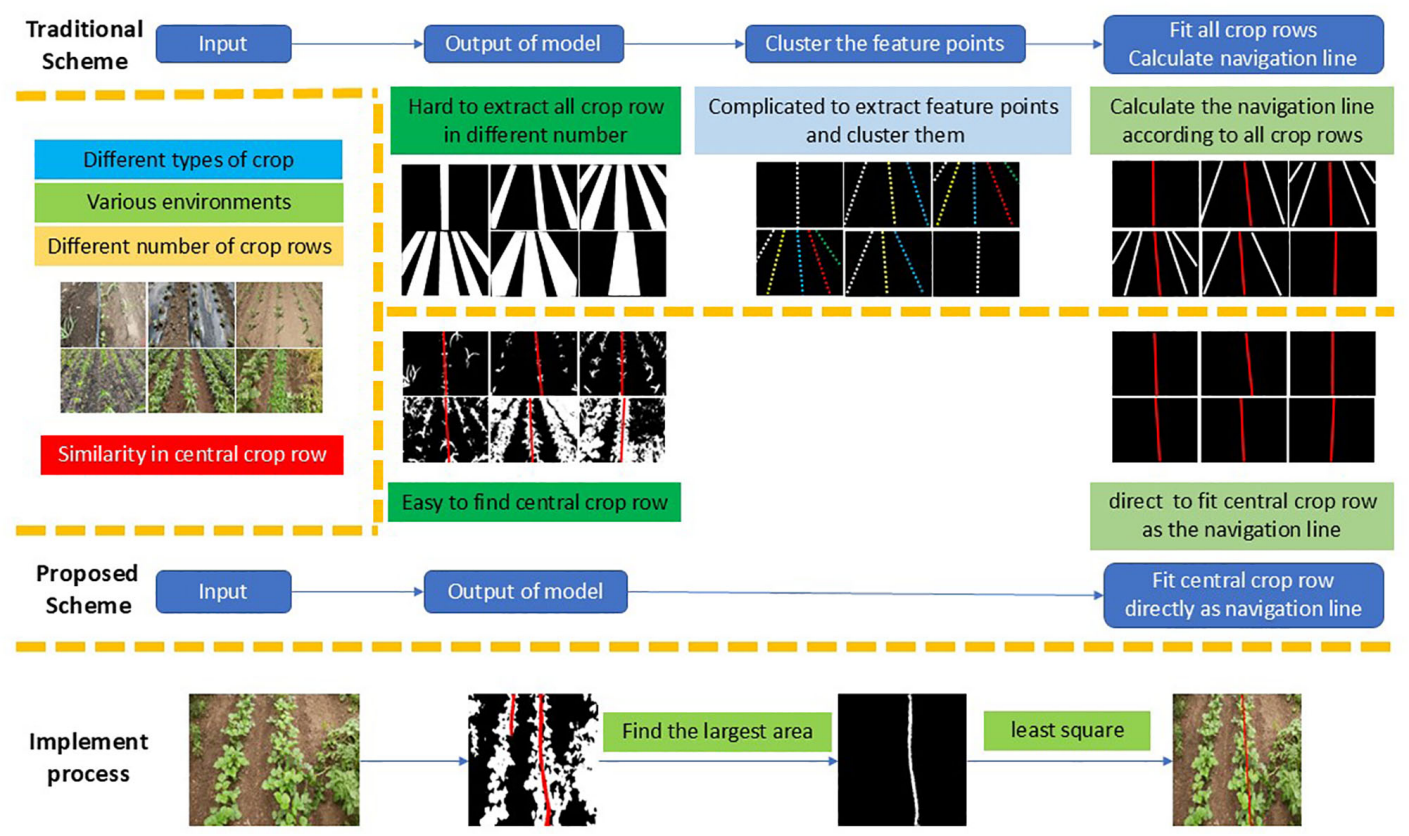
Comparison between the traditional multi-stage navigation line extraction framework and the proposed central crop row detection scheme. The conventional approach (top) relies on semantic segmentation, feature point extraction, clustering, and line fitting to infer the navigation line, while the proposed method (bottom) directly detects the central crop row and performs least-squares fitting to obtain the navigation line, resulting in a more streamlined and robust process.

Currently, most semantic segmentation-based navigation line extraction methods follow a unified multi-stage framework, as illustrated in **Figure 1**. In this pipeline, segmentation models are first used to detect all crop rows, followed by feature point extraction for each row, clustering (e.g., K-means), and line fitting to obtain the final navigation line. However, a single model often struggles to accurately detect crop rows under diverse field scenarios (such as the six images in **Figure 1**). Furthermore, variations in the number of visible crop rows can disrupt downstream processing, causing incorrect clustering or erroneous region assignments. These factors severely limit the robustness and generalization of such systems in real-world deployments.

However, despite the diversity of field environments, a consistent spatial prior can be observed across all six scenes shown in **Figure 1**: crops are planted in a highly regular pattern, and a central crop row is always present. This row naturally serves as the primary visual reference for human operators when steering agricultural machinery. Motivated by this observation, we propose a streamlined paradigm that abandons redundant multi-row inference and instead focuses solely on detecting the central crop row, treating it directly as the navigation line.

Specifically, a semantic segmentation network is trained to output three pixel-wise classes: background, vegetation (green region), and the central crop row (highlighted in red). To further avoid handcrafted post-processing and manual parameter tuning, we simply select the largest connected component within the line class and perform least-squares fitting to obtain the final navigation line.

Compared with traditional schemes, the proposed method collapses multiple algorithmic stages into an almost single end-to-end formulation. The reliability of navigation line estimation depends directly on the model output, without relying on handcrafted rules to adapt to changing crop structures or environmental conditions. This design ensures superior stability, stronger transferability, and minimal engineering overhead during deployment.

### 2.2 The architecture of CCRDNet

**Figure 2:**
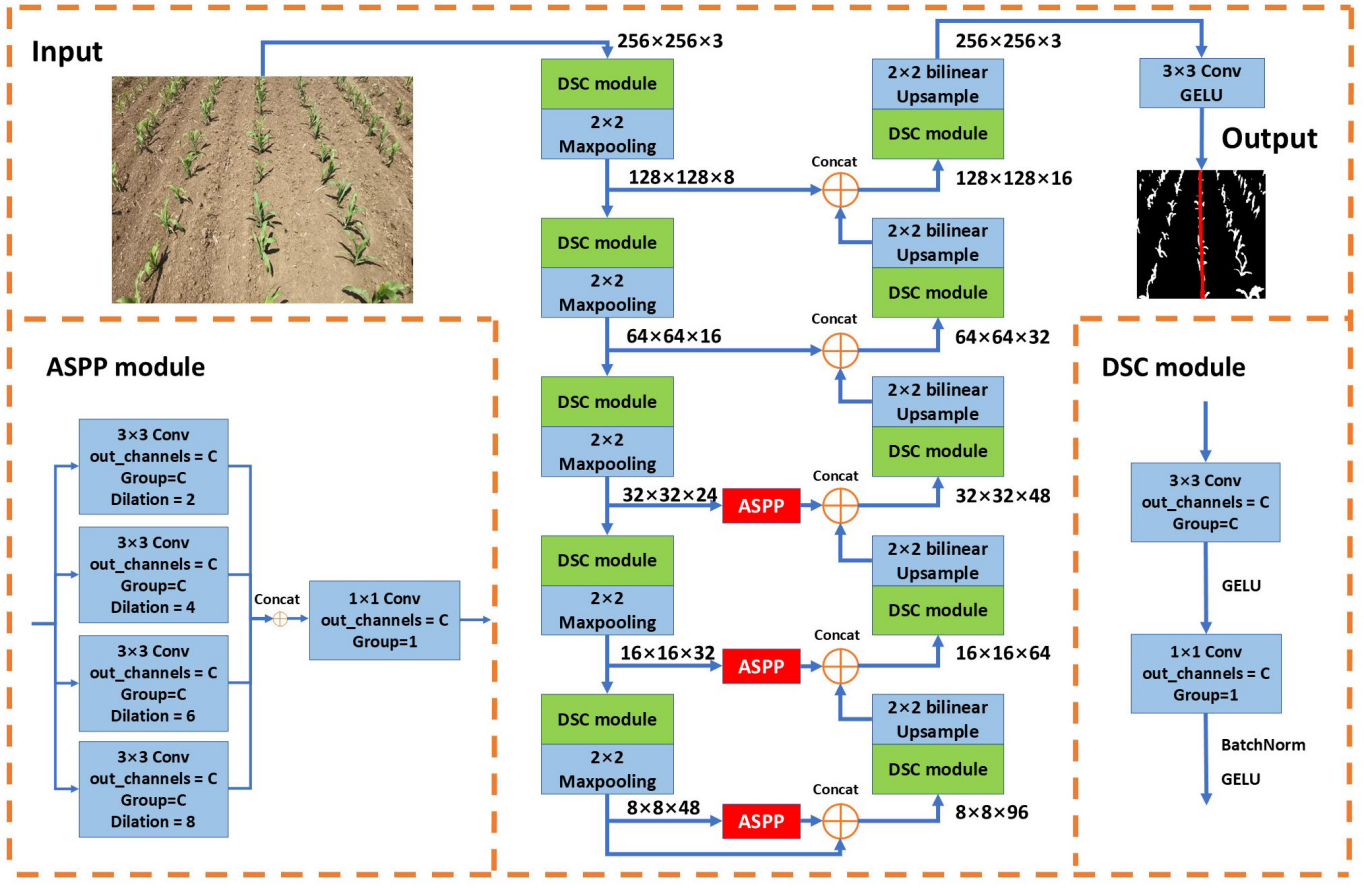
Overall architecture of the proposed Central Crop Row Detection Network (CCRDNet). The model follows a U-shaped encoder–decoder structure with skip connections, incorporating depthwise separable convolution (DSC) blocks for lightweight feature extraction and Atrous Spatial Pyramid Pooling (ASPP) modules for multi-scale context aggregation. The output is a pixel-wise prediction map containing background, vegetation, and central crop row classes.

The lightweight model, Central Crop Row Detection Network (CCRDNet), is proposed specifically for the task addressed in this paper. UNet (Ronneberger et al., 2015) is widely recognized as a highly effective architecture for semantic segmentation, due to its U-shaped design and skip connections that integrate features from downsampling and upsampling stages. Inspired by this, CCRDNet also adopts a U-shaped architecture with skip connections, as illustrated in **Figure 2**.

To meet the real-time requirements of agricultural deployment, the standard convolutional blocks in UNet are replaced with depthwise separable convolution (DSC) modules (Chollet, 2016). Each DSC module consists of a 3×3 grouped convolution followed by a 1×1 pointwise convolution, with a GELU activation applied between them. Batch normalization and GELU are further applied at the end of the module. This design substantially reduces the number of parameters while maintaining strong feature extraction capabilities, supporting the lightweight and high-speed objectives of CCRDNet.

Given that the task focuses solely on extracting the central crop row, precise identification is critical, particularly under challenging scenarios such as rotated or shifted rows, occlusion by nearby weeds, or non-contiguous crop sections. To address these challenges, we incorporate an Atrous Spatial Pyramid Pooling (ASPP) module (Chen et al., 2018), which employs multiple dilated convolutions with varying rates to capture features at multiple receptive fields. This enables the network to learn multi-scale contextual information, essential for linking separated crops and accurately detecting the central row.

In our design, the ASPP module also employs grouped convolutions to reduce parameters. Four dilated convolutions with rates of 2, 4, 6, and 8 are applied, and their outputs are concatenated and passed through a 1×1 convolution to adjust channel dimensions. To balance feature preservation and efficiency, the ASPP modules are incorporated only into the last three skip connections. This arrangement allows the network to retain original image details during downsampling, while simultaneously emphasizing individual crop features in low-level representations and capturing inter-crop connectivity in high-level features during upsampling. By effectively combining low- and high-level semantic information, this design ensures precise detection of the central crop row.

### 2.3 Navigation line fitting method

After semantic segmentation, the navigation line must be derived from the predicted line mask. Although several techniques such as the RANSAC algorithm and Hough transform are available, the least-squares method is adopted in this study due to its simplicity and computational efficiency. The line parameters are computed with Equations 1, 2 as follows.

(1) $$\theta =\frac{\sum_{i=1}^{n}(x_{i}y_{i})-n\bar{x}\;\bar{y}}{\sum_{i=1}^{n}x_{i}^{2}-n{\bar{x}}^{2}}$$

(2) $$b=\bar{y}-\theta \bar{x}$$

However, the least-squares method is inherently sensitive to outliers; directly using all pixels classified as the line category can lead to inaccurate fitting when misclassifications occur. To enhance robustness and maintain universality without manual parameter tuning, the navigation line is fitted based on the largest connected component within the predicted line class, as illustrated in **Figure 1**. This strategy ensures that only the most reliable portion of the detected central crop row contributes to line fitting, effectively mitigating the impact of spurious or fragmented regions.

### 2.4 Dataset description

To enable universal detection of the central crop row across diverse planting conditions, a large-scale dataset comprising 7,367 annotated images was collected. The dataset covers eight crop species, including 3,324 maize, 798 eggplant, 1,575 soybean, 388 mung bean, 301 pepper, 400 scallion, 181 carrot, and 400 tomato samples. All images were captured using a handheld mobile phone (Honor V30 Pro) in Harbin, Heilongjiang Province, China (45.7567°N, 126.6424°E). To ensure visual diversity, no constraints were imposed on camera height or shooting angle, resulting in rich variations in scale, orientation, and lighting. The original resolutions of 1280×720 and 1920×1080 were uniformly resized to 640×480 before annotation.

Each image was manually verified to ensure the presence of a central crop row, with varying degrees of tilt and positional deviation to simulate real-world navigation scenarios. In addition to crop variety, the dataset intentionally incorporates environmental disturbances such as shadows, soil reflections, lighting variability, and weed occlusion, making it highly representative of practical field conditions.

**Figure 3:**
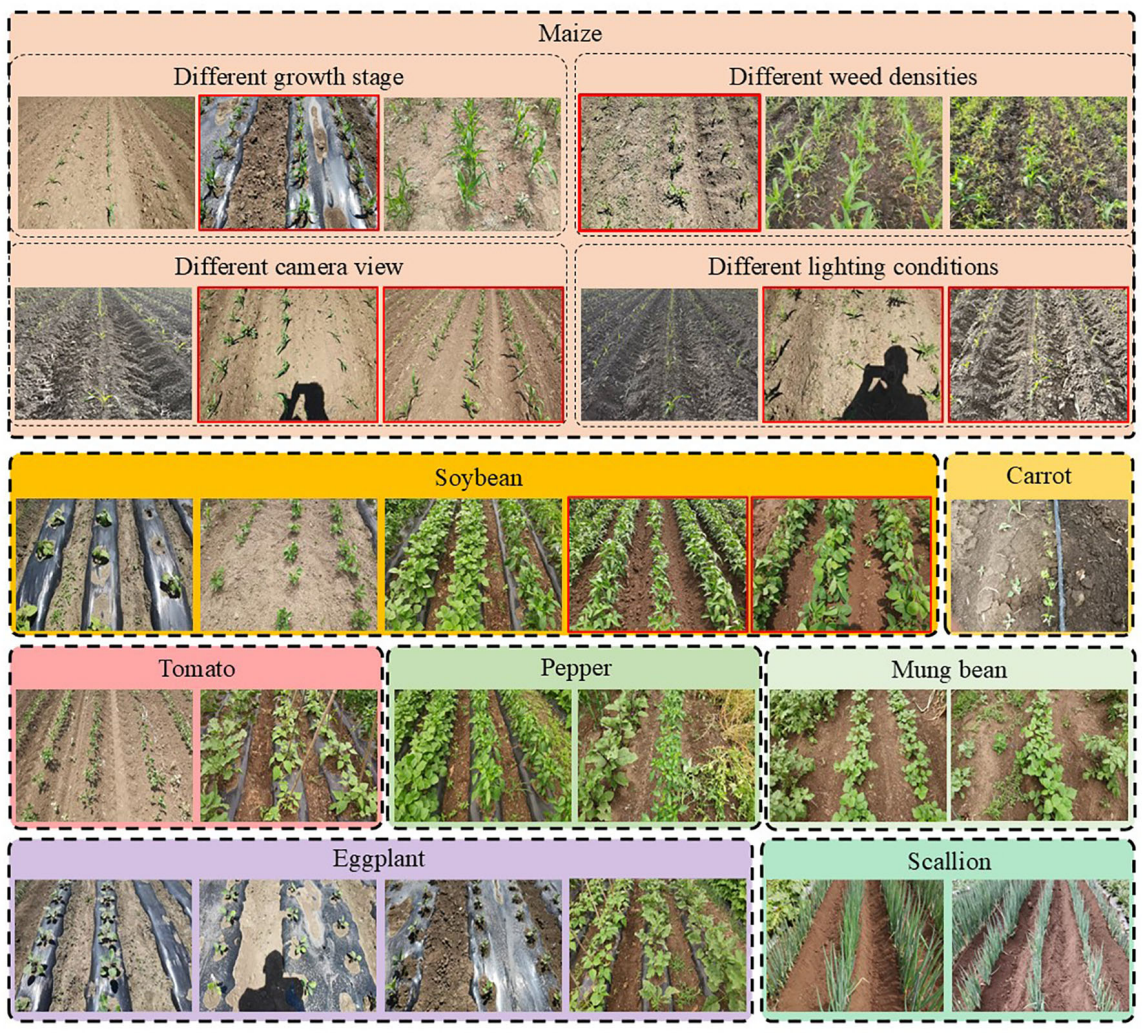
Overview of the proposed dataset and its diversity across different agricultural conditions. The dataset covers multiple crop species and a wide range of field environments. Maize is presented as a representative example to illustrate dataset diversity, including different crop growth stages, varying weed densities, multiple camera viewpoints, and diverse illumination conditions. Images highlighted with red bounding boxes are used for training, while the remaining images correspond to zero-shot scenarios that were not observed during training.

The overall dataset is illustrated in **Figure 3**. In addition to covering a wide variety of crop species, the dataset demonstrates its diversity through a categorized visualization at the top of the figure, where maize serves as a representative example. Specifically, the dataset encompasses crops at different growth stages, varying weed densities, multiple camera viewpoints, and diverse illumination conditions. Images highlighted with red bounding boxes are used for model training, while the remaining images correspond to zero-shot scenarios that were entirely unseen during the training phase. Overall, the dataset exhibits high richness and variability, providing a challenging and comprehensive benchmark for evaluating the robustness, generalization capability, and real-world applicability of in-row navigation methods under diverse agricultural conditions.

### 2.5 Annotation strategy

**Figure 4:**
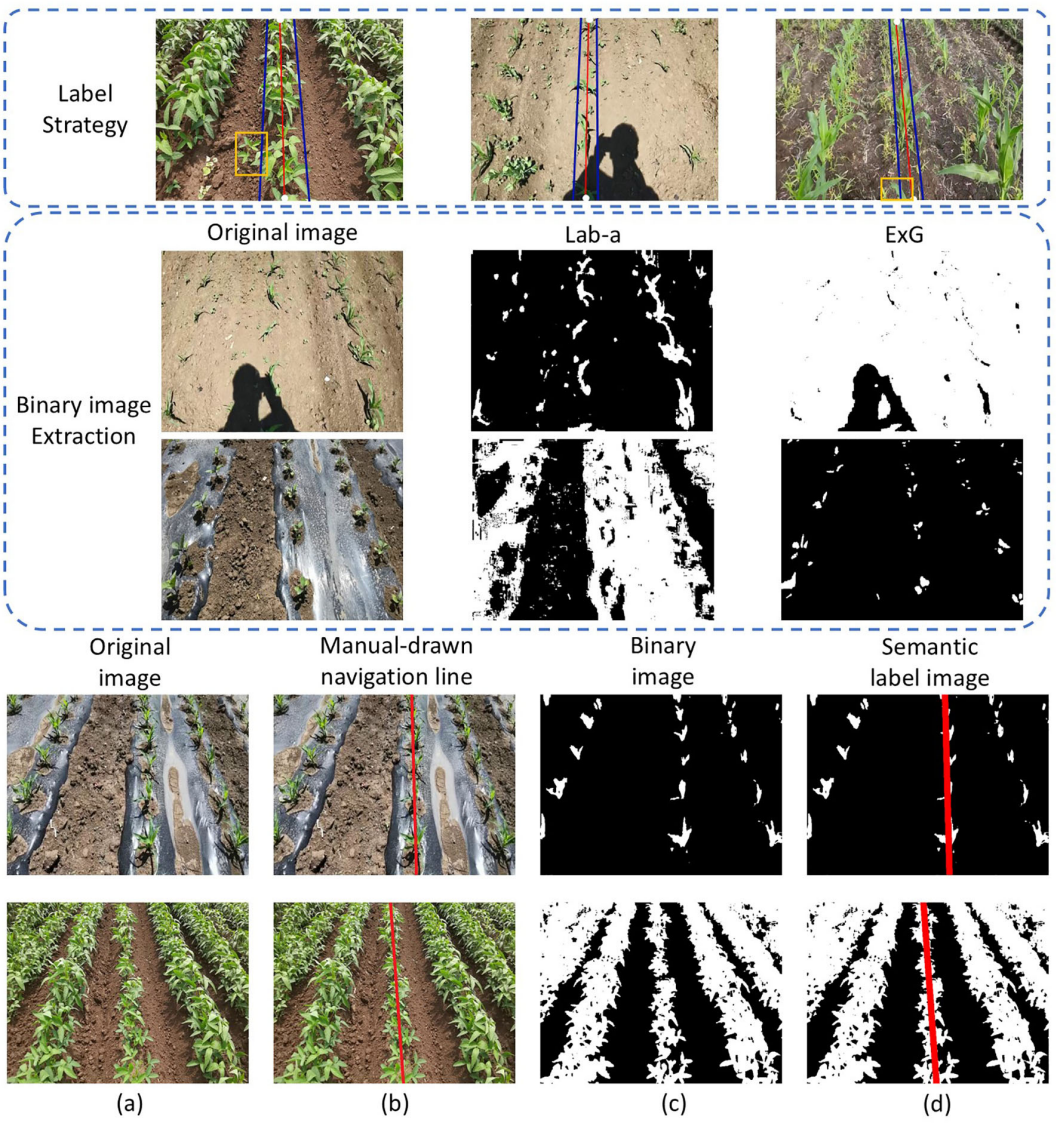
Annotation strategy and binary image selection. Blue lines indicate crop row boundaries, white dots mark selected midpoints, and the red line denotes the annotated central crop row. Leaves within the yellow boxes are ignored. Binary images of vegetable class were generated using either Lab-a or ExG, depending on environmental robustness. **(a)** Original image; **(b)** Image with manually drawn navigation line; **(c)** Binary image; **(d)** Semantic label image with three classes: background, vegetation, and navigation line (15 pixels width).

**Figure 4** illustrates the annotation workflow used to construct the dataset. In precision agriculture, the goal of crop row detection is to extract navigation guidelines for autonomous vehicles rather than to perform pixel-perfect segmentation of every individual plant. Real crop rows naturally exhibit minor irregularities due to individual plant variations, leaf occlusions, and missing plants, but the underlying row structure—the line that a robot should follow—remains conceptually straight under most field conditions. This is particularly true for our dataset, which contains almost no crop rows with significant curvature. Therefore, since the vast majority of collected images contain predominantly straight crop rows, the central row was annotated using a single straight line, which also serves as the navigation reference.

The annotation process involves two main steps: first, the approximate boundaries of the target row are identified (blue lines), and then the midpoints at the upper and lower image edges are selected (white dots) to determine the final navigation line (red line). Although some leaves within the yellow boxes appear slightly misaligned, such local irregularities do not affect the overall determination of the central crop row, as they represent natural variations rather than fundamental deviations in row structure.

In parallel, binary masks were generated using traditional vegetation extraction algorithms. Two commonly used indices were employed: Excess Green (ExG) (Woebbecke et al., 1995) and the a-channel of the Lab color space (Lab-a) (Riehle et al., 2020). ExG is calculated by Equation 3 as:

(3) ExG=2G−R−B

where *R*, *G*, and *B* are the red, green, and blue components of the RGB image, respectively. For the Lab-a method, the image is converted to the Lab color space, the a-channel is extracted as a grayscale image, and Otsu thresholding is applied to obtain the binary result. Neither method is universally robust under all environmental conditions (see **Figure 4**), so the final binary image for each sample was manually selected, prioritizing Lab-a and using ExG when Lab-a failed.

Unlike conventional approaches that segment all crop rows indiscriminately, our annotation strategy explicitly distinguishes the central crop row as a dedicated target. Specifically, the vegetation region is annotated as one class, while the central crop row is assigned to a separate class. As a result, the ground truth consists of three semantic categories: background, vegetation, and the central crop row (navigation line).

This annotation design is motivated by two key considerations. First, it follows a human-like visual perception pattern in which crops are naturally distinguished from the soil background, and the central crop row is then identified as the primary reference for navigation. Second, by introducing an explicit representation of the central crop row, the vegetation class serves as an auxiliary supervisory signal that provides structural constraints to guide the model’s localization process. This design is particularly important given that only the central crop row region is relevant for navigation, while background pixels dominate the majority of each image. The additional vegetation class helps mitigate class imbalance and prevents the model from overfitting to background features.

In agronomic terms, a crop row represents the planting line—ideally, the centerline connecting crop root positions—rather than the variable extent of foliage. The apparent visual width of crop rows changes dramatically across growth stages, from sparse seedlings to dense canopies, but the underlying geometric structure remains constant. Annotating variable widths would conflate plant morphology with row geometry, introducing unnecessary intra-class variation that could confuse the model. To maintain consistency and provide the model with a stable learning target, a fixed width of 15 pixels was uniformly assigned to all annotated navigation lines. This width is sufficient to provide a clear learning signal while ensuring that the annotation represents the structural essence of the crop row rather than its transient visual appearance.

### 2.6 Evaluation indicators

The proposed task consists of two stages: detecting the central crop row using the segmentation network and fitting the corresponding navigation line. To comprehensively evaluate both the segmentation accuracy and the quality of line fitting, six metrics were adopted.

1. Mean Intersection over Union (mIoU): mIoU quantifies segmentation performance by averaging the Intersection over Union (IoU) across all classes. IoU is defined as the ratio between the overlapping area of the predicted and ground-truth masks and their union.
2. Pixel Accuracy (PA): PA denotes the proportion of correctly classified pixels relative to the total number of pixels.
3. Angle Error (AE): AE measures the angular deviation between the fitted navigation line and the manually annotated reference.
4. Line Intersection over Union (L_IoU): Line_IoU (L_IoU) evaluates the spatial similarity between the fitted navigation line and the ground truth, with both represented as 15-pixel-wide masks. It is computed as the standard Intersection over Union (IoU) with Equation 4:

(4) $$L\_\text{IoU}=\frac{{\text{Area}}_{\text{intersection}}}{{\text{Area}}_{\text{union}}}$$

To interpret this metric in terms of practical navigation accuracy, we note that since both lines have a fixed width of 15 pixels, an L_IoU of 50% corresponds to an overlapping width of approximately 10 pixels. This translates to a lateral offset of only 5 pixels between the two centerlines, which we consider an acceptable tolerance for autonomous navigation.

5. Line Accuracy (LA): LA further assesses the reliability of the fitted line. A navigation line is regarded as correct if its angular deviation satisfies *AE <* 5^°^ and *L*_IoU_*>* 50%.
6. Frames Per Second (FPS): FPS measures computational efficiency by reporting the number of frames processed per second.

In summary, mIoU and PA are employed to evaluate the segmentation model output, whereas AE, L_IoU, LA, and FPS are dedicated to assessing the performance of navigation line fitting.

## 3 Experiments and results

### 3.1 Data augmentation and training environment

**Figure 5:**
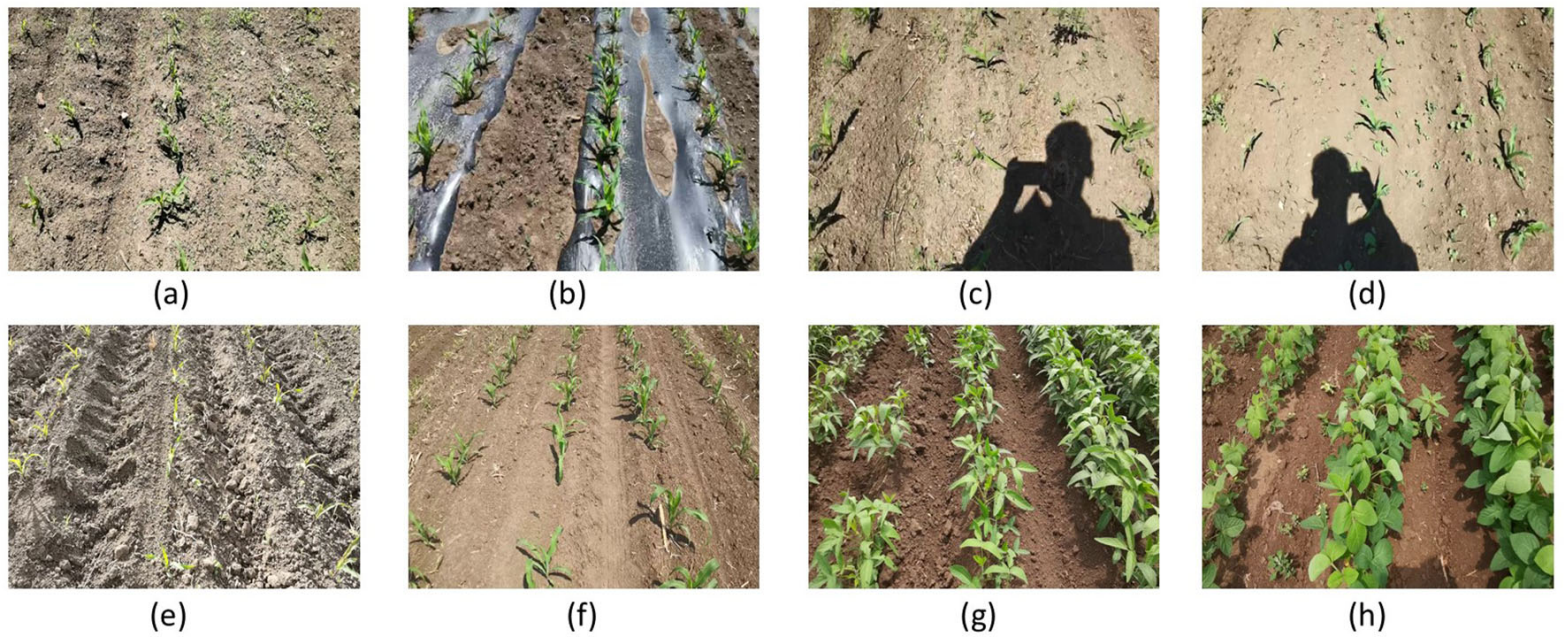
Eight representative scenarios in the training set. Images **(a–f)** depict maize at different growth stages and environmental conditions, while **(g, h)** show soybean with continuous crop structures. The dataset includes diverse illumination, weed density, and background variations.

For training, images were selected from eight distinct environments, as illustrated in **Figure 5**. The training set encompasses two crop types and incorporates diverse challenges such as weed interference, reflective mulch films, shadows, and illumination variations. Images (a)–(f) depict maize at different growth stages under various interference conditions, representing discrete crop structures, while images (g) and (h) correspond to soybean fields, illustrating continuous crop row patterns. A total of 50 images were sampled from each environment, yielding 400 training samples—accounting for only 5.4% of the entire dataset. This configuration enables a rigorous evaluation of the model’s data efficiency and generalization capability under limited supervision.

Considering the actual motion patterns of agricultural vehicles, crop rows in real fields are generally straight but often appear with slight inclination in captured images. Even curved rows can be locally approximated as a combination of straight and slanted segments. To emulate these variations and improve model robustness, random rotation was applied to all training and testing samples. This operation ensures that the model learns to handle a wide range of angular deviations caused by camera orientation or vehicle attitude changes.

In addition, lateral displacement of the central crop row within the camera’s field of view was simulated by applying vertical cropping from either the left or right side of the image, effectively mimicking the visual effect of small steering offsets during navigation. However, extreme transformations—such as excessive rotation or overly aggressive lateral cropping—would correspond to unrealistically large steering deviations that typically require manual correction in practical field operations. Therefore, the augmentation range was constrained to ±40°for rotation and ±60 pixels for lateral cropping, ensuring geometric plausibility while introducing sufficient variability.

To maintain strict separation between training and evaluation data, identical augmentation strategies were applied to the test set without reusing or duplicating any original training images. In total, the dataset consisted of 7,367 images used exclusively for testing, while 400 images were dedicated to training after augmentation. This procedure ensured that no overlap existed between the two sets and that the evaluation reliably reflected the model’s genuine generalization capability.

All dataset construction, model training, and inference procedures were conducted on a Lenovo Legion R9000P laptop equipped with an NVIDIA GeForce RTX 3060 GPU and an AMD Ryzen 7 5800H CPU with Radeon Graphics. To evaluate the model’s real-world deployability on embedded platforms, the trained network was also tested on an NVIDIA Jetson Orin NX with 8GB RAM.

The network was trained for 500 epochs using input images with 256×256 resolution and a batch size of 4. The Adam optimizer was employed with an initial learning rate of 0.0002 and momentum coefficients *β*_1_ = 0.5 and *β*_2_ = 0.999. Cross-entropy loss was adopted as the training objective.

### 3.2 Results and analysis

**Table 1** summarizes the overall performance of CCRDNet across the full test set containing 7367 images. When evaluated on the entire dataset without distinction, the model achieves a Pixel Accuracy (PA) of 96.02%, a mean Intersection over Union (mIoU) of 73.72%, and a Line IoU (*L_IoU*) of 78.37%, with an average Angle Error (AE) of only 1.13°. More than 95% of the samples satisfy the accuracy criterion (*AE <* 5° and *L_IoU >* 50%), demonstrating strong reliability.

**Table 1:** Performance of CCRDNet on the full dataset. “Seen” refers to environments included in the training set, while “Unseen” indicates zero-shot environments with variations in crop types, illumination, and planting patterns.

Model	Seen	Number	PA(%)	mIoU(%)	L_IoU (%)	AE(°)	LA(%)
	–	7367	96.02	73.72	78.37	1.13	95.57
CCRDNet	✓	2200	97.59	80.91	85.47	0.71	99.54
	×	5167	95.35	70.66	75.35	1.31	93.88

To further assess the model’s generalization capability, the dataset was divided into *seen* and *unseen* environments according to their presence in the training set. In seen scenarios, CCRDNet achieves near-perfect performance, with PA and *L_IoU* reaching 97.59% and 85.47%, respectively, and an exceptionally low AE of 0.71°. Even in unseen environments—featuring different crops, lighting conditions, and planting structures—the model maintains competitive performance, achieving 95.35% PA and 75.35% *L_IoU*. The slight degradation is expected due to domain variations, yet the model still preserves a high Line Accuracy (LA) of 93.88%, confirming its strong transferability to previously unseen field conditions.

These quantitative results validate the effectiveness of directly detecting the central crop row using a lightweight segmentation network. To gain deeper insight into its behavior across different conditions, representative visual examples are provided in the following section for qualitative analysis.

#### 3.2.1 No weeds in different environments

Under ideal or near-ideal field conditions with minimal weed interference, the main challenges arise from variations in crop type, growth stage, planting density, and lighting. Even in these relatively simple scenarios, inconsistencies such as missing plants, irregular spacing, or uncertain number of crop rows can lead to unstable extraction when using traditional algorithms.

**Figure 6:**
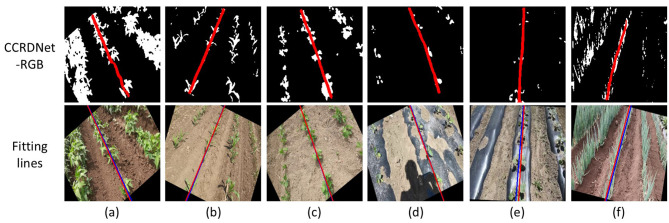
Outputs of CCRDNet and corresponding navigation line fitting results under weed-free or low-interference conditions. Blue lines indicate the manually annotated ground truth, and red lines denote the navigation lines predicted by CCRDNet. **(a, b)** Soybean and maize fields with noticeable gaps along the central row. **(c)** Soybean at a later growth stage with strong rotation. **(d)** Eggplant on reflective ground film with human shadows. **(e)** Two-row configuration with missing left row. **(f)** Scallion field with curved central row under intense illumination.

As shown in **Figure 6**, CCRDNet consistently detects and follows the central crop row with high precision. The fitted navigation lines align closely with the manually annotated references across diverse crop and illumination conditions. In each image, the blue line represents the ground-truth annotation, while the red line denotes the predicted central crop row obtained by CCRDNet, which is also treated as the navigation line.

Images (a)–(f) cover four crop types across different growth stages and illumination levels. Specifically, (a) and (b) show soybean and maize fields, respectively, both containing visible gaps along the central crop row. Image (c) depicts soybean at a later growth stage with notable rotation. Image (d) shows eggplant grown on reflective ground film under strong illumination with human shadows. Image (e) illustrates a two-row configuration with a missing left row, and (f) presents five scallion rows on red soil under intense lighting, where the central row is slightly curved and off-centered.

These examples exhibit considerable variation in crop morphology, spacing, and environmental disturbance (e.g., shadows, ground film, or overexposure). Nevertheless, the proposed model maintains robust performance across all cases, successfully tracking the true central crop row and producing fitted navigation lines closely aligned with the annotated ground truth.

#### 3.2.2 Weeds around a clear central crop row

**Figure 7:**
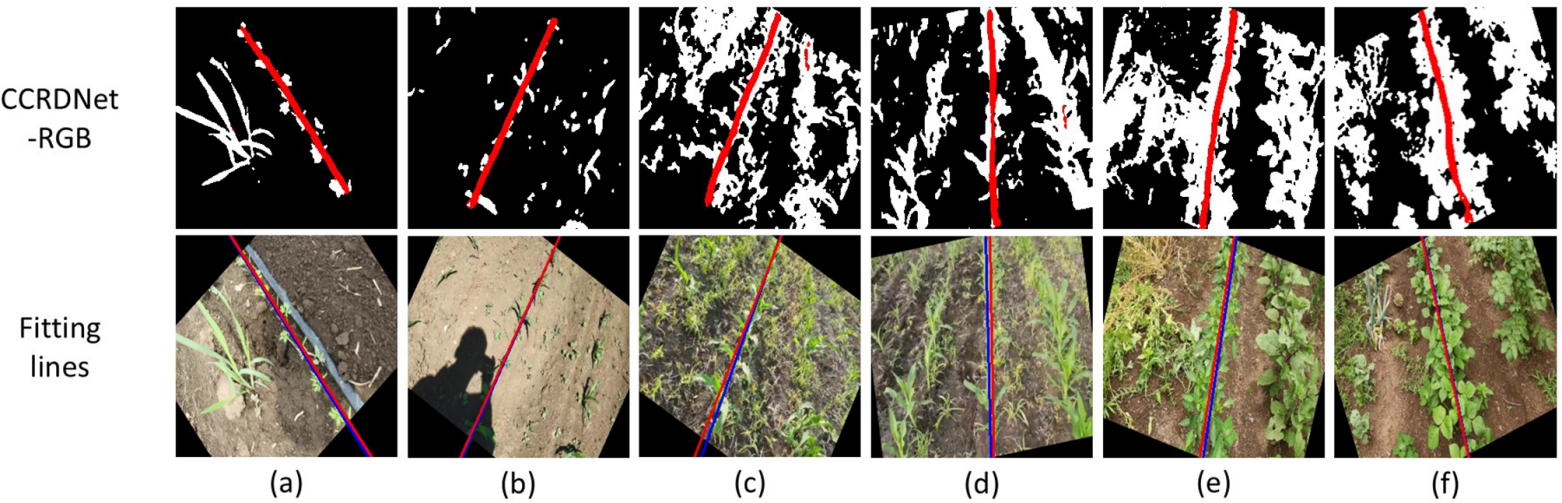
Outputs of CCRDNet and corresponding navigation line fitting in weed-contaminated environments where the central crop row remains visually distinguishable. **(a)** Weeds larger than carrot seedlings. **(b)** Scattered weeds with human shadow interference. **(c, d)** Dense weeds intertwined with crops. **(e, f)** Highly complex scenes with diverse weed species and mixed crop types. Only **(b)** appears in the training set, while the remaining cases represent unseen environments.

In scenarios with significant weed presence, central crop row detection becomes substantially more difficult due to irregular weed distribution, the similar appearance between weeds and crops, and occlusions that disrupt row continuity. Classical vision-based approaches often misidentify weeds as crops or fail to isolate the true row structure. As illustrated in **Figure 7**, CCRDNet effectively suppresses weed interference and reliably tracks the central crop row even in highly cluttered and chaotic environments, demonstrating strong robustness against biological noise.

**Figures 7a–f** illustrate several weed-contaminated scenarios, among which only (b) belongs to the training set. In **Figure 7a**, the weeds are noticeably larger than the carrot seedlings. In (b), numerous weeds are densely distributed on both sides of the central crop row, with sizes comparable to the crops, making it infeasible to suppress weed interference using size thresholds or erosion-based processing.

**Figures 7c, d** present even more challenging conditions, where weeds are widely spread and intertwined with the crops, rendering the central crop row visually indistinguishable and preventing efficient weed removal. Moreover, (e) and (f) depict highly complex environments containing multiple weed species with diverse morphologies and coexisting crop types.

Such uncertainty in weed distribution, combined with the irregular number and positions of crop rows, poses serious challenges to traditional extraction algorithms. Likewise, the coexistence of different crop types within a single image may confuse models trained for a specific crop species. Despite these difficulties, the central crop row remains visually distinguishable, and CCRDNet successfully captures it across all cases, demonstrating strong robustness and generalization ability in weed-dense environments. Furthermore, the fitted navigation lines closely align with the labeled ground truth, confirming the reliability of the model even under severe visual interference.

#### 3.2.3 Performance on curved crop row

Curved or heavily slanted crop rows pose another significant challenge for vision-based navigation systems, particularly when models are primarily trained on datasets dominated by straight-row patterns. Since our collected dataset mainly consists of nearly straight crop rows, random rotation was applied during both training and testing to synthetically simulate slanted or inclined row structures. As previously discussed, CCRDNet maintains high robustness across a wide range of inclination angles, suggesting its ability to adapt to more complex geometries.

**Figure 8:**
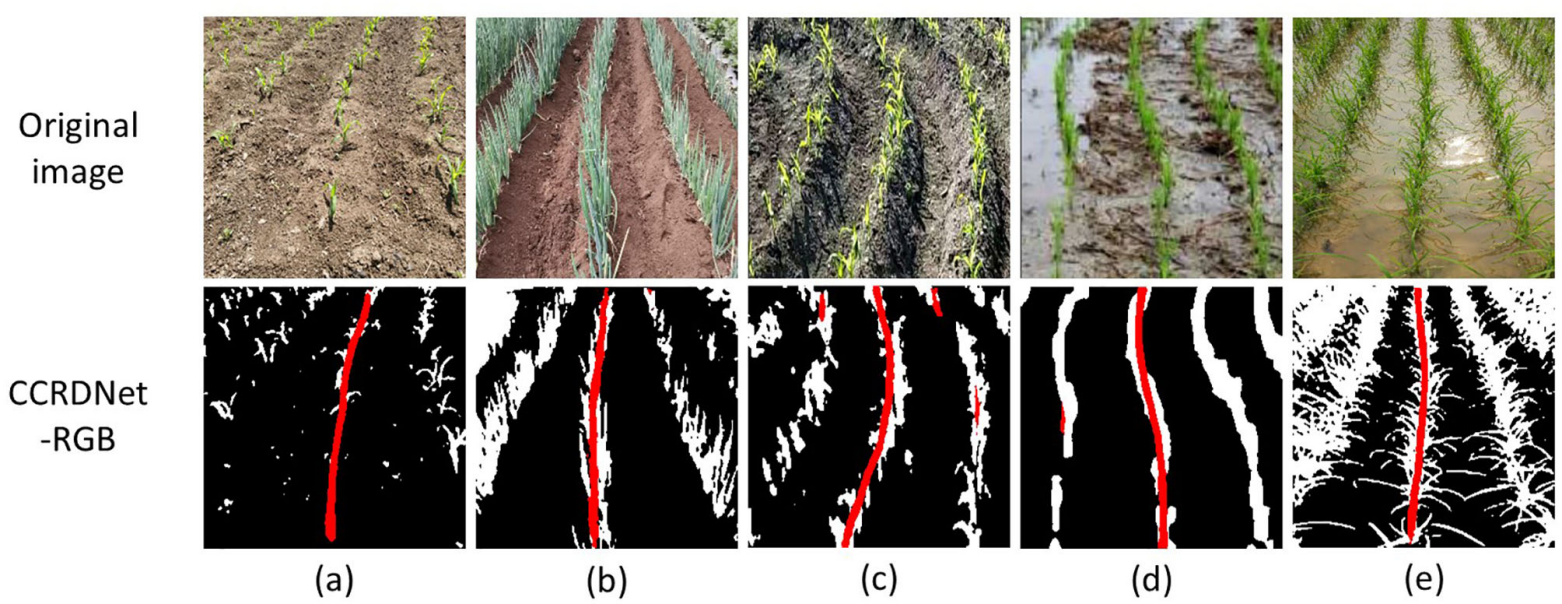
Outputs of CCRDNet and corresponding navigation line fitting on curved crop rows. **(c)** is from Guo et al. (2024); **(d)** is provided by the author Wang et al. (2023); **(f)** is sourced from a public GitHub dataset (https://github.com/liu-zey/public-dataset/tree/master).

Beyond these synthetic transformations, CCRDNet also demonstrates strong capability in processing genuinely curved crop rows. As shown in **Figure 8**, the model successfully follows the continuous curvature of the central crop row even when evaluated on external datasets that were never involved in training. This finding indicates that the proposed approach not only performs reliably under ideal straight-row conditions but also generalizes effectively to irregular or non-linear field structures.

### 3.3 Impact of architectural components

To evaluate the effectiveness of the proposed architectural components, ablation experiments were conducted on the Depthwise Separable Convolution (DSC) and the Atrous Spatial Pyramid Pooling (ASPP) module. The results are summarized in **Table 2**.

**Table 2:** Ablation experiments on CCRDNet architecture.

Model	DSC	ASPP	Flops	Parameters	AE(°)	L_IoU(%)	LA(%)	FPS-1-G
CCRDNet-1	X	X	100.844M	77.186K	1.32	75.73	91.71	101.179
CCRDNet-2	X	✓	141.967M	235.530K	1.09	78.97	95.85	80.45
CCRDNet-3	✓	X	31.791M	12.077K	1.43	74.30	90.06	105.04
CCRDNet	✓	✓	38.226M	33.621K	1.13	78.37	95.57	86.76

DSC denotes Depthwise Separable Convolution, and ASPP denotes Atrous Spatial Pyramid Pooling. FPS-1-G refers to experiments performed on the laptop using GPU (RTX3060).

Replacing standard convolutions with DSC (*CCRDNet-3* vs. *CCRDNet-1*) led to a 68.5% reduction in FLOPs and an 84% decrease in parameters, while maintaining comparable fitting accuracy. This confirms that the navigation line fitting framework effectively simplifies the task, enabling the use of an extremely lightweight backbone without sacrificing performance.

Introducing the ASPP module (*CCRDNet-2* vs. *CCRDNet-1*) significantly boosted line fitting accuracy, reducing AE from 1.32° to 1.09° and increasing LA by over 4%, albeit at the cost of additional computation. When combined with DSC (*CCRDNet*), ASPP achieved an ideal trade-off, yielding the highest accuracy (95.57% LA) with only a 14% speed reduction compared to the baseline.

Overall, the DSC primarily contributes to computational efficiency, while the ASPP module brings substantial gains in accuracy. Their combination forms a balanced solution that aligns with the dual objective of real-time performance and high-precision navigation. Based on these findings, both DSC and ASPP are retained in the final *CCRDNet* design, ensuring an optimal trade-off between model efficiency and accuracy.

### 3.4 Comparison experiments

To ensure fair comparison, all models below were trained from scratch on our dataset using identical configurations. We used the Adam optimizer with learning rate 0.0002 and momentum coefficients *β*_1_ = 0.5 and *β*_2_ = 0.999. Models were trained for 500 epochs with batch size 4 and input resolution 256×256. Cross-entropy loss was used as the objective function.

#### 3.4.1 Comparison with multi-row extraction

To further validate the necessity of explicitly targeting only the central crop row, we conducted comparative experiments using ENet Paszke et al. (2016) and MS_ERFNet Liu et al. (2023). ENet is a lightweight semantic segmentation network, while MS_ERFNet is specifically designed for crop row extraction in agricultural scenes. Both models were trained under two annotation settings: (1) datasets annotated with only the central crop row, and (2) datasets annotated with all visible crop rows. For a fair comparison, the same 400 images were used in both settings, and the training protocol was kept identical. Evaluation was performed on the entire dataset.

**Figure 9:**
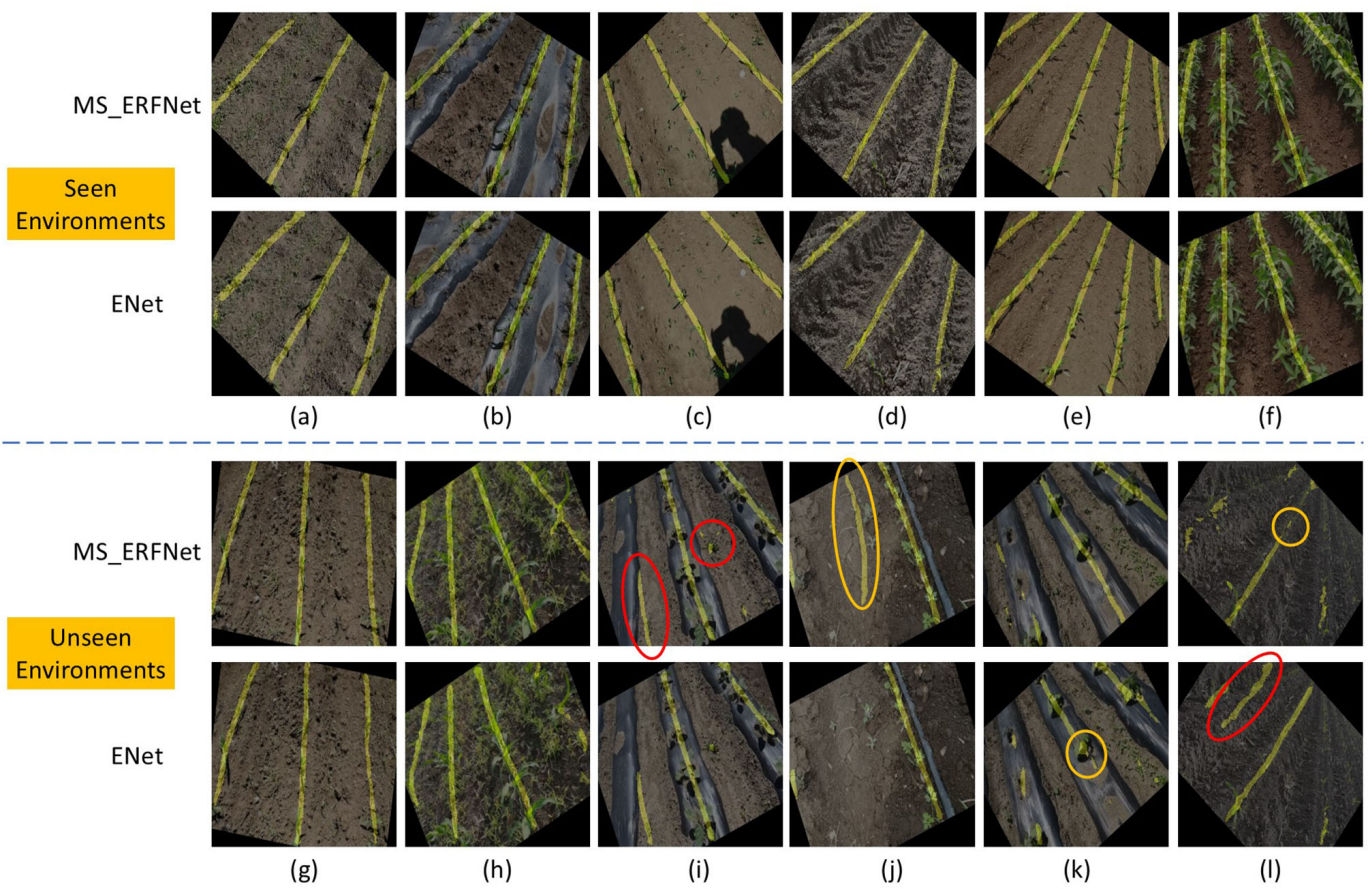
Segmentation outputs of MS_ERFNet and ENet when extracting all crop rows in different environments (shown as white regions). Images **(a–f)** represent training scenarios, while images **(g–l)** are from unseen environments. Red circles indicate regions where no actual crop rows exist but are erroneously predicted by the models; yellow circles highlight unstable or missing outputs in regions that would influence navigation line fitting.

Representative qualitative results are shown in **Figure 9**. Images (a)–(f) correspond to environments that were seen during training, where both models are able to successfully extract all visible crop rows, despite being trained with only 50 samples per category. However, when evaluated on unseen environments, the segmentation performance degrades noticeably. When crop rows are visually clear and evenly distributed, both models can still correctly detect multiple rows, as illustrated in (g) and (h). In contrast, when the number or spatial distribution of crop rows changes, erroneous detections frequently occur, as shown in (i)–(l).

Notably, even when the models fail to correctly detect lateral crop rows, the central crop row is often preserved with reasonable fidelity. This observation supports our hypothesis that explicitly constraining the task to “central crop row only” reduces ambiguity caused by variations in row count and spatial layout, thereby enabling the network to focus on navigation-critical information.

We further investigated whether reliable navigation lines could be fitted from the outputs of all-row extraction models. In practice, inconsistent numbers of detected rows and frequent prediction artifacts make it challenging to design a universal rule-based post-processing strategy for feature point extraction and clustering. Given the structural characteristics of crop row distributions, a simple heuristic baseline was adopted: selecting the connected component whose centroid is closest to the image center.

The quantitative results are summarized in **Table 3**. For both MS_ERFNet and ENet, training with annotations explicitly targeting only the central crop row consistently outperforms the all-row annotation strategy across all evaluation metrics. Specifically, MS_ERFNet achieves a 0.79% improvement in L_IoU, a 0.22° reduction in angular error (AE), and a 1.06% increase in line accuracy (LA) when using center-only supervision. ENet exhibits even larger performance gains, with L_IoU increasing by 2.75%, AE decreasing by 0.20°, and LA improving by 1.02%.

**Table 3:** Quantitative comparison of different annotation strategies on navigation performance.

Model	Strategy	L_IoU (%)	AE (°)	LA (%)
MS_ERFNet	All crop rows	76.83	1.39	92.76
	Center crop row	77.62	1.17	93.82
ENet	All crop rows	74.35	1.45	91.63
	Center crop row	77.10	1.25	92.65
CCRDNet	Center crop row	78.37	1.13	95.57

As illustrated in **Figure 9**, regions highlighted by red circles correspond to incorrect or unstable predictions that do not directly contribute to navigation line fitting, whereas areas marked by yellow circles indicate regions actually used for navigation line estimation. Although some erroneous detections appear visually irrelevant, their presence introduces additional noise and uncertainty into the fitting process within different strategy, ultimately degrading both the robustness and reliability of the resulting navigation lines.

Overall, these results demonstrate that segmenting all crop rows introduces unnecessary complexity for navigation-oriented perception tasks, particularly under varying field conditions. In contrast, explicitly supervising the central crop row provides a task-aligned inductive bias that yields more stable predictions and more reliable navigation line estimation.

#### 3.4.2 Comparison with two categories annotation

**Figure 10:**
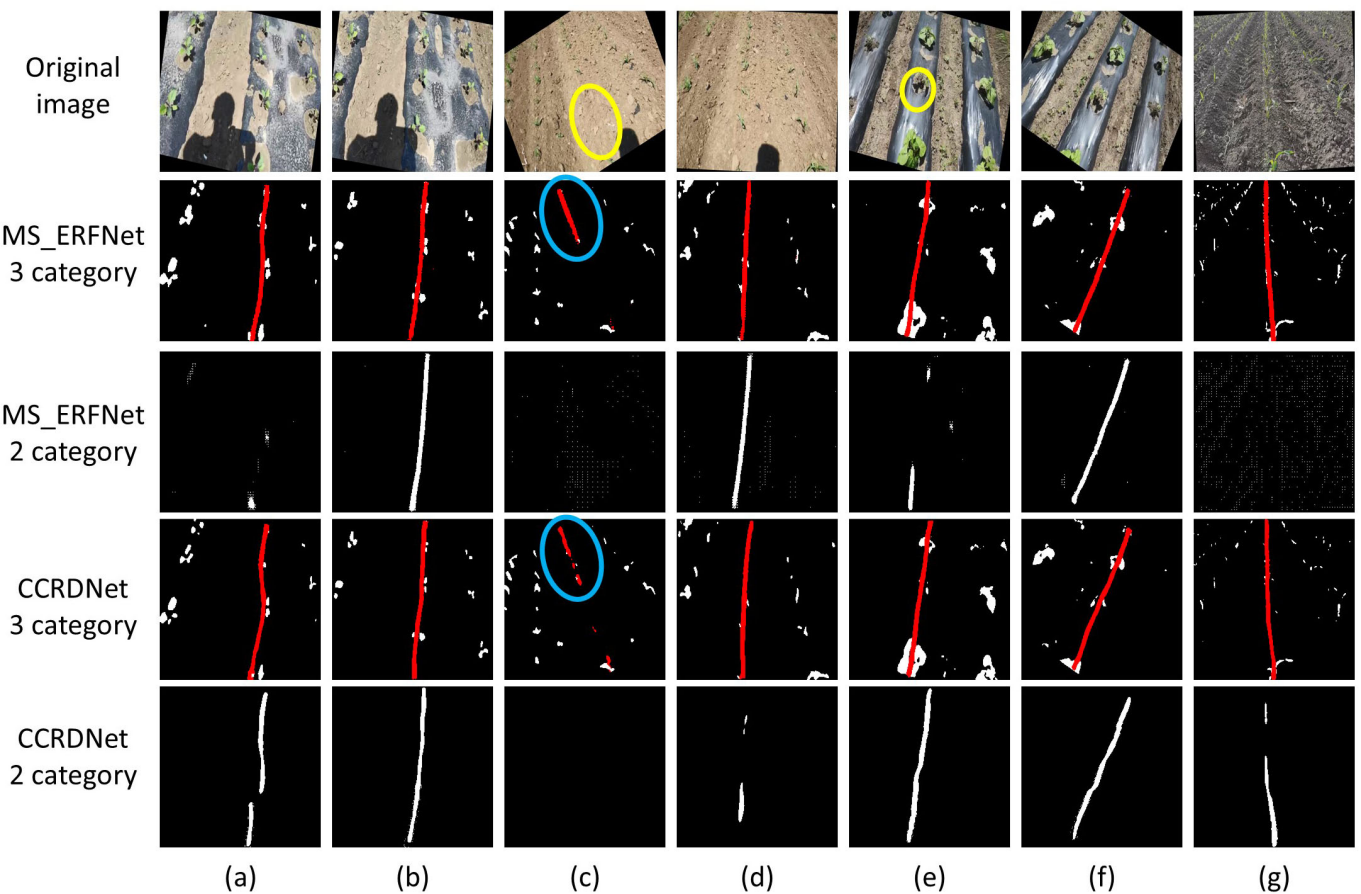
Qualitative comparison of detection performance between two-class (without vegetation) and three-class (with vegetation) training schemes. **(a)** Scenario characterized by uneven illumination; **(c, e)** challenging scenarios with missing crops; **(g)** a scene captured from a shifted camera viewpoint. Yellow circles highlight regions where crops are absent, causing potential localization ambiguity. Blue circles demonstrate the robust predictions of the three-class model, which leverages the spatial context of the vegetation category to maintain accurate row detection.

To further investigate the significance of incorporating an explicit vegetation category, comparative experiments were conducted using MS_ERFNet and CCRDNet. Both models were trained on a consistent set of 400 images under two configurations: a three-class scheme and a two-class scheme (where the vegetation class was removed, leaving only the background and the central crop row). Representative qualitative results in previously unseen environments are illustrated in **Figure 10**.

In scenarios where crop rows are well-defined and uniformly distributed, both configurations yield satisfactory results. However, the two-class models frequently fail under challenging conditions, such as uneven illumination (**Figure 10a**), missing crops (yellow circles), or fluctuating camera viewpoints (**Figure 10g**). Conversely, models integrated with vegetation supervision successfully infer the central crop row’s position by leveraging the spatial distribution of the surrounding vegetation (blue circles).

This performance disparity stems from the lack of intermediate semantic structure in the two-class approach. Without the vegetation category, all non-navigation regions are collapsed into a single “background” class, hindering the model’s ability to learn the structural morphology of crop rows. Consequently, the network becomes overly reliant on global appearance cues, making it susceptible to overfitting and environmental noise—a vulnerability exacerbated by severe class imbalance.

By explicitly introducing vegetation as a distinct category, the annotation scheme aligns more closely with human visual perception—distinguishing crops from soil before identifying specific navigation lines. Crucially, vegetation segmentation serves as an auxiliary supervisory signal that provides structural constraints, guiding the localization of the central row.

As demonstrated in **Table 4**, the three-class setting consistently enhances navigation metrics (higher L_IoU and LA, lower angular error), confirming that vegetation-aware supervision is vital for stabilizing crop row detection in complex agricultural landscapes.

**Table 4:** Effect of introducing a vegetation category on navigation performance.

Model	Categories	L_IoU (%)	AE (°)	LA (%)
MS_ERFNet	2	75.31	1.56	91.47
	3	77.62	1.17	93.82
CCRDNet	2	74.92	1.38	91.46
	3	78.37	1.13	95.57

#### 3.4.3 Comparison with other models

For comprehensive comparison, except for ENet and MS_ERFNet, another two representative models were selected as baselines: SegNet Badrinarayanan et al. (2017) and ResC-UNet Zhang et al. (2023). SegNet utilizes max-pooling index recording to reduce memory overhead during upsampling, while ResC-UNet is task-specific networks originally designed for crop row detection.

**Figure 11:**
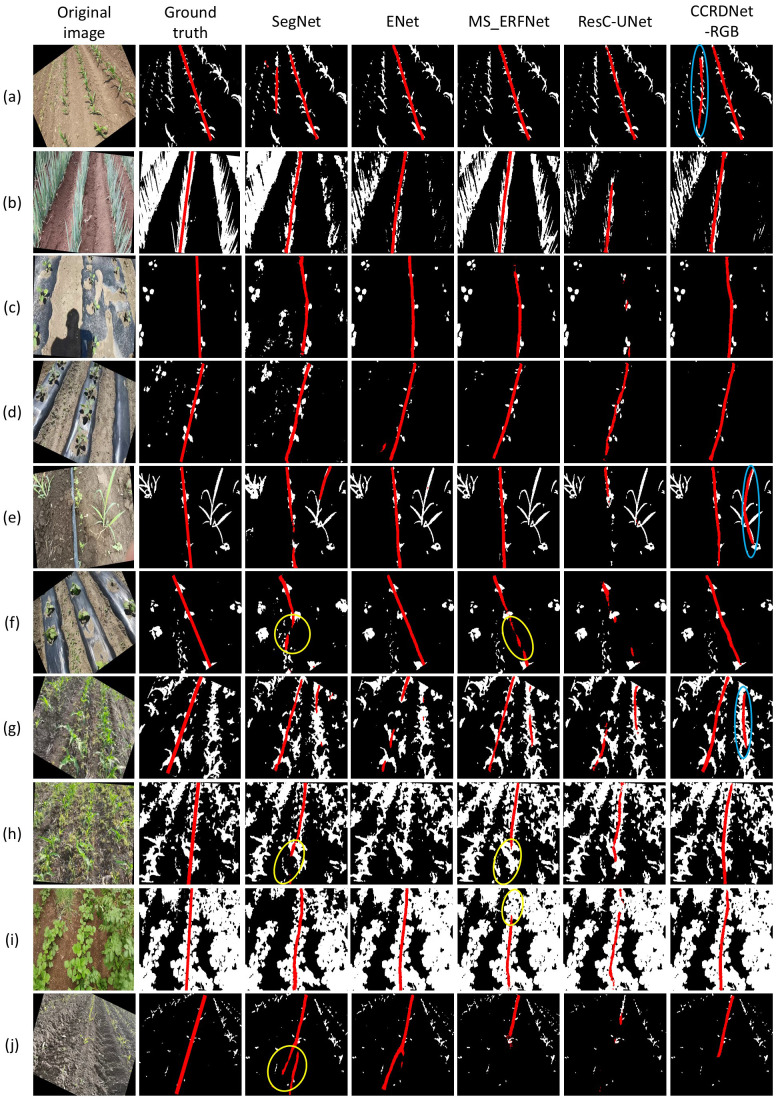
Visual outputs of different models. Images **(a–d)** correspond to clean environments without weeds; **(e–h)** depict varying weed interference; **(i)** shows a complex environment; **(j)** has missing crops in the bottom region. Yellow circles highlight regions where certain models lack detail, and blue circles indicate prediction errors outside the central crop row.

**Figure 11** illustrates the visual outputs across diverse field conditions. Images (a)–(d) correspond to clean environments without weeds, (e)–(h) contain varying degrees of weed occlusion, (i) involves mixed crop types, and (j) presents a challenging scenario with missing crops in the lower part of the image. Across all cases, CCRDNet consistently locates the central crop row with high precision. Slight weaknesses appear under extremely strong illumination ((b) and (d)), which is typical for RGB-based segmentation methods.

ResC-UNet performs the worst, likely due to its excessive parameter count, while ENet suffers from over-segmentation in heavy-weed conditions ((g) and (h)). MS_ERFNet and SegNet produce blurred or incomplete boundaries in several regions (highlighted by yellow circles).

**Table 5** provides a quantitative comparison of the models across segmentation and navigation metrics. Although MS_ERFNet achieves superior segmentation performance (PA = 96.52%, mIoU = 75.56%), CCRDNet outperforms all other models in navigation line extraction, yielding the lowest angle error (1.13°), the highest line IoU (78.37%), and the best line accuracy (95.57%).

**Table 5:** Comprehensive comparison of CCRDNet and baseline models in terms of accuracy, robustness, and computational efficiency.

Model	Performance metrics					Efficiency metrics					
	PA (%)	mIoU (%)	L_IoU (%)	AE (°)	LA (%)	GFLOPs	Params (M)	FPS-1-G	FPS-1-C	FPS-2-G	FPS-2-C
SegNet	94.66	70.98	77.12	1.28	93.72	40.207	29.431	48.44	1.39	15.63	0.73
ENet	95.96	74.05	77.10	1.25	92.65	0.542	0.349	37.39	12.82	20.24	**3.03**
MS_ERFNet	**96.52**	**75.56**	77.62	1.17	93.82	2.478	7.157	57.16	25.45	25.91	1.35
ResC-UNet	95.32	67.15	66.18	2.72	76.23	99.496	130.534	34.54	2.27	8.78	0.68
CCRDNet	96.02	73.72	**78.37**	**1.13**	**95.57**	**0.038**	**0.033**	**86.76**	**40.94**	**48.37**	2.58

FPS-1 and FPS-2 denote FPS performance on laptop and Jetson Orin NX; C and G indicate CPU and GPU, respectively. Bold body representation is the optimal result.

The comparatively lower PA and mIoU of CCRDNet relative to MS_ERFNet warrant further examination. As illustrated in **Figure 11**, the difference primarily stems from two factors: minor semantic misclassifications of lateral crop rows (indicated by blue circles), and less precise segmentation boundaries within the vegetation class. However, these pixel-level inaccuracies in non-central regions do not compromise the model’s primary objective—accurate localization of the central crop row for navigation. MS_ERFNet’s higher segmentation scores reflect its ability to delineate all visible crop structures, but this comes at the cost of reduced robustness and increased complexity in extracting the navigation-critical centerline. The small training set (400 images) may exacerbate this tendency, as models attempting to learn more complex representations (all rows) are more susceptible to overfitting compared to those focused on a single, well-defined target (central row only). CCRDNet’s architecture effectively concentrates learned features on the navigation line itself, resulting in more stable and accurate line fitting despite marginally lower pixel-wise segmentation metrics.

Model efficiency was evaluated in terms of parameter size and frame rate on both a laptop and a Jetson Orin NX development board (**Table 5**). CCRDNet is the lightest model with only 0.033 M parameters and 0.038 GFLOPs, achieving 86.76 FPS on laptop GPU and 48.37 FPS on Orin NX GPU without TensorRT. In contrast, ResC-UNet, with over 130 M parameters, fails to reach real-time inference, highlighting the importance of lightweight design for practical agricultural deployment.

In summary, CCRDNet provides the best balance between accuracy and efficiency, enabling stable, real-time navigation line extraction suitable for onboard deployment in field robots.

## 4 Discussion

### 4.1 Methodological innovation and generalization

In this study, we introduced effective innovations in both detection strategy and annotation design. By explicitly tailoring the detection approach to the navigation task—focusing solely on extracting the central crop row rather than all visible rows—we demonstrated that the overall algorithmic framework can be significantly simplified without compromising performance.

Although crop row detection employs semantic segmentation models, it fundamentally differs from conventional pixel-level segmentation tasks. This distinction necessitates task-specific adaptations in annotation strategy, including: (1) uniform straight-line annotation to represent the underlying row structure rather than variable foliage extent, (2) introduction of a vegetation class to provide auxiliary supervision and structural constraints, and (3) consistent annotation width across all samples to enable the model to learn core structural characteristics independent of growth stage variations.

Remarkably, training on only 400 images from eight distinct crop environments yielded strong generalization performance. This capability extends beyond central crop row detection, models trained on our dataset with multi-row annotation still exhibit commendable performance in zero-shot multi-row extraction scenarios when crop rows are uniformly distributed and clearly defined. These findings suggest that in the context of crop row detection, the sheer volume of data is not the primary determinant of performance. Instead, enhancing dataset diversity—allowing the model to comprehensively learn from varied environmental conditions—is paramount. This perspective motivated our decision to publicly release the dataset. We anticipate that by strategically increasing the environmental complexity of training data, the efficacy of these methods can be further enhanced.

### 4.2 Limitations

**Figure 12:**
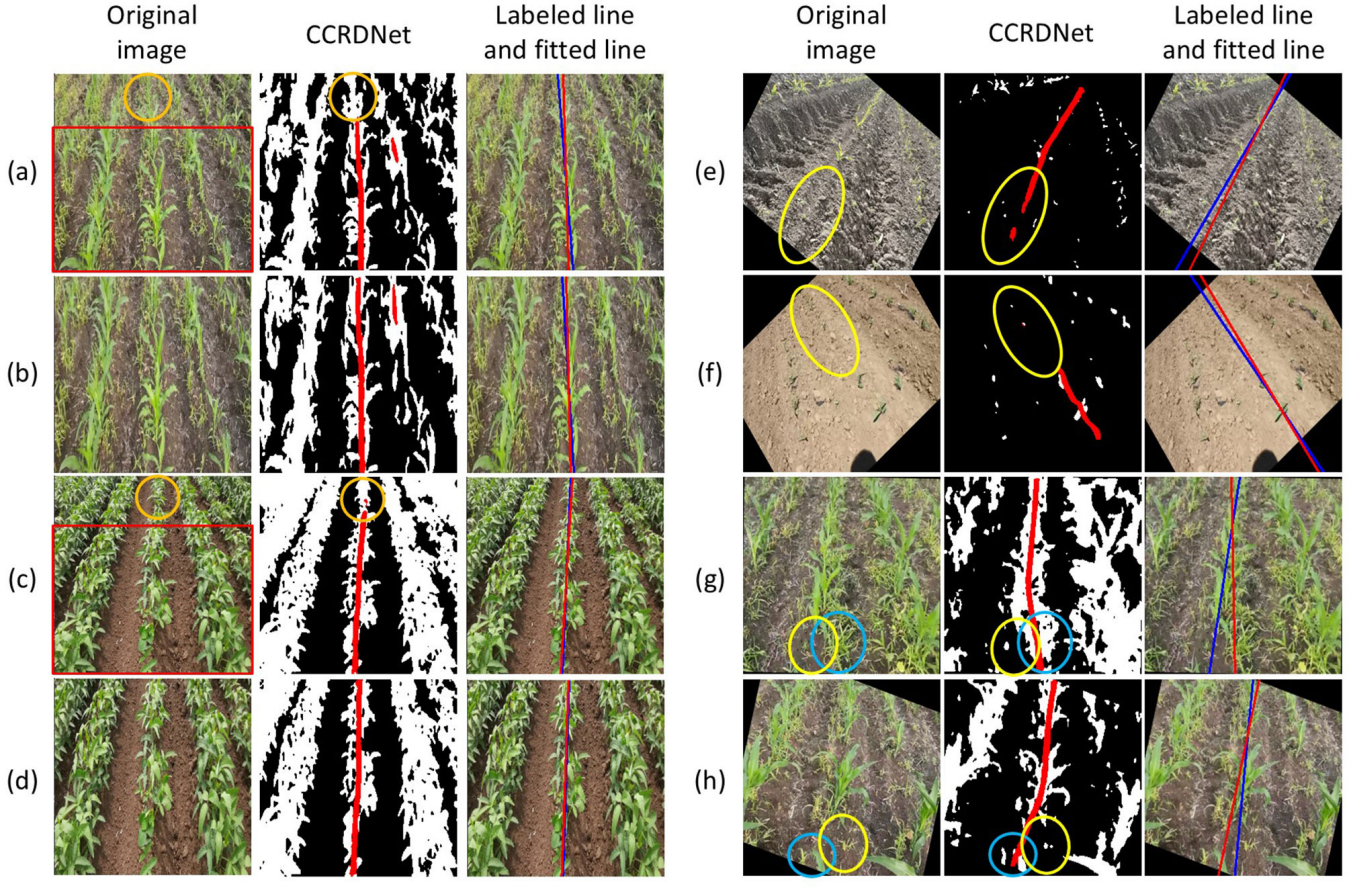
Limitations of the proposed CCRDNet model. **(a, c)** show scenarios with densely arranged crop rows, while **(b, d)** present their cropped versions with improved performance. **(e–h)** illustrate challenges in handling crop absence. Yellow circles highlight large areas of missing crops; blue circles emphasize the confounding effects of weeds and crop leaves surrounding the gaps.

Despite the promising performance of CCRDNet, several limitations remain to be addressed, as illustrated in **Figure 12**.

Dense crop row arrangements. One primary challenge arises in environments where crop rows are densely arranged, which typically occurs during late growth stages or when the camera captures multiple rows at steep viewing angles. In such conditions, the visual separation between adjacent rows becomes blurred, making it difficult for the model to accurately distinguish the central crop row, particularly in distant image regions. This ambiguity occasionally causes navigation line deviations when excessive peripheral vegetation is included in the field of view. Empirically, restricting the region of interest to the central area of the image can mitigate this issue by reducing interference from non-target rows and enhancing the spatial consistency of detected features, as shown in the comparison between images (a) and (b), and (c) and (d) in **Figure 12**.

Missing crops along the central row. Because CCRDNet fundamentally relies on the continuity of visible crop features, large gaps—caused by missing seedlings, mechanical damage, or weed occlusion—can disrupt the estimated navigation trajectory, as shown in images (e)–(h). When small discontinuities occur, the model can often infer the missing portions through contextual cues from surrounding vegetation. However, extended interruptions, particularly when combined with dense weed interference or irregular soil textures, may lead to instability or inaccurate line fitting. This phenomenon highlights a fundamental challenge faced by most vision-based navigation systems: the inherent variability and structural incompleteness of real agricultural environments.

Overall, CCRDNet performs most reliably when images contain a moderate number of well-defined rows (typically three to five) and when crop structures exhibit reasonable spatial continuity. Addressing these limitations represents important directions for future research to ensure more robust and consistent navigation across the full spectrum of field conditions encountered in practical agricultural operations.

### 4.3 Potential extension: binary input representation

**Figure 13:**
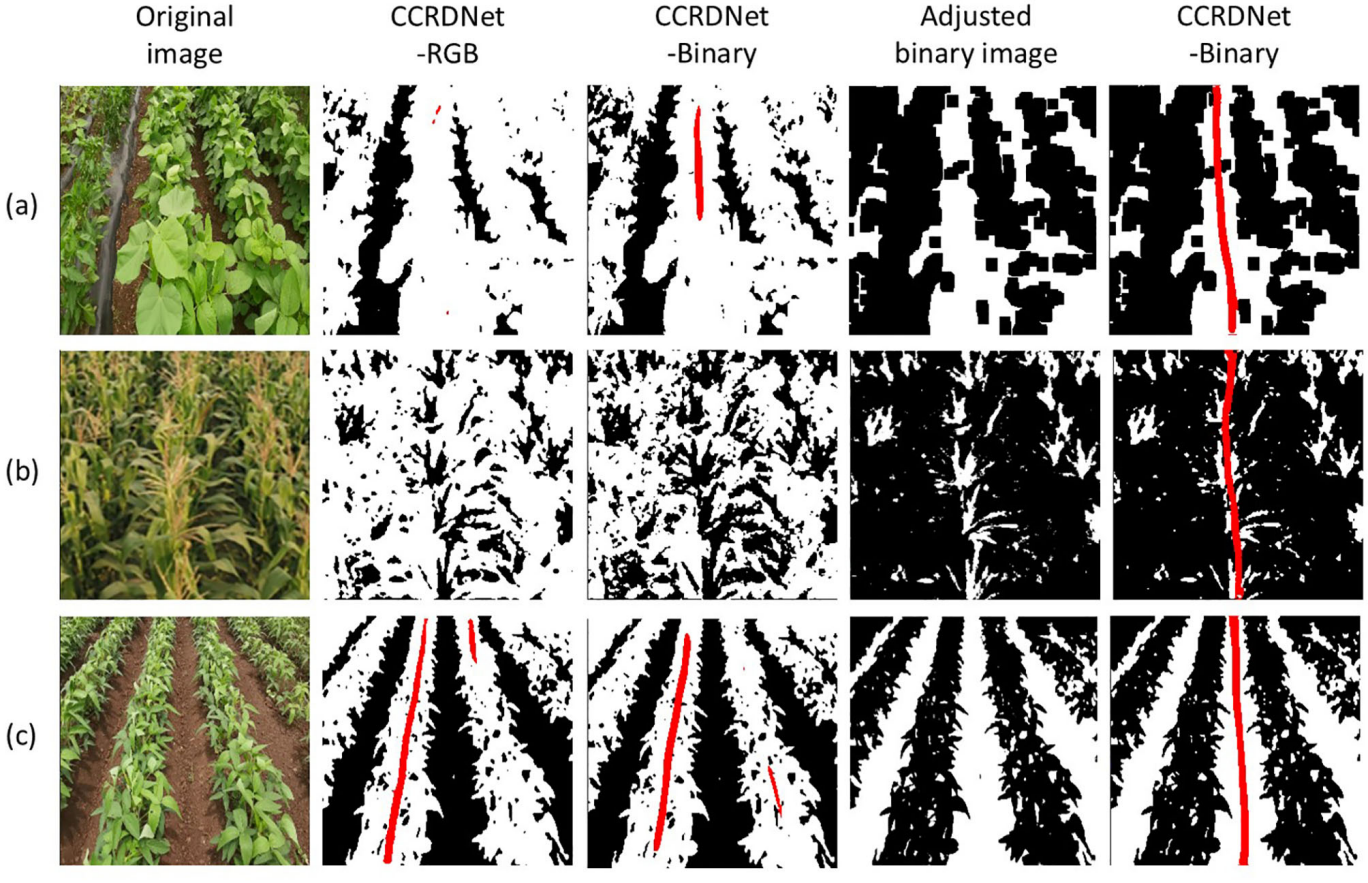
**(a)** illustrates a late growth stage in which crop rows nearly overlap, making the central crop row indistinguishable for conventional models. By applying morphological erosion to refine the binary image, CCRDNet-Binary successfully identifies the central row. In **(b)**, where maize tassels dominate the image, traditional color-based segmentation fails to identify meaningful row features. By isolating yellow components in the HSV space, a binary mask representing tassel positions enables accurate row detection. Finally, **(c)** presents symmetric crop rows, where standard models tend to misidentify side rows as the center. When represented in binary form, CCRDNet-Binary correctly aligns with the true central trajectory, demonstrating its robustness under symmetric conditions. These preliminary findings indicate that simplifying input representation may further enhance generalization across crop types and growth stages.

In this study, to enhance the generalization capability of the model, we focus on directly detecting the central crop row. The results demonstrate that the model performs well across various crop types and growth stages. Since the core objective is to localize the central crop row rather than rely on specific crop color, size, or type, we further explore the use of binary image inputs, which retain only spatial information of crops and thus may improve model generality and environmental invariance. This abstraction enables CCRDNet to focus purely on structural cues of crop rows. Preliminary experiments with the binary-input variant, termed CCRDNet-Binary, show that it can effectively handle challenging cases such as overlapping crop rows, late growth stages, tasseling maize, and symmetric row configurations (**Figure 13**).

### 4.4 Future work

While the proposed method demonstrates strong performance across diverse scenarios, several promising directions warrant further investigation to address current limitations and extend the method’s applicability.

Dataset expansion and distribution refinement. The current training set, though small (400 images), already achieves notable generalization through strategic sampling of diverse environmental conditions. However, as identified in Section 4.2, certain challenging scenarios—such as densely arranged crop rows during late growth stages—remain underrepresented. Expanding the dataset to include more samples from these edge cases, while maintaining balanced coverage of various crop species, growth stages, and interference factors, could further enhance model robustness. Specifically, increasing representation of high-density row configurations, extreme occlusion scenarios, and transitional growth stages would enable the model to learn more discriminative features for boundary cases.

Handling discontinuous crop rows. As demonstrated in Section 4.2, the model’s performance degrades when the central crop row exhibits significant gaps due to missing plants or severe occlusion. Incorporating temporal information from video sequences represents a promising solution, enabling the system to maintain tracking continuity by leveraging motion cues and historical observations across frames. When a gap is detected in the current frame, the system could propagate the navigation line from previous frames using predictive models such as Kalman filtering, ensuring smooth trajectory estimation even during temporary occlusions. Alternatively, hybrid approaches that combine segmentation-based detection with geometric priors—such as enforcing spatial consistency constraints or learning gap-filling strategies from complete row structures—could provide more robust predictions. Another potential direction involves exploring attention mechanisms that explicitly model long-range dependencies along the row direction, allowing the network to “bridge” missing segments by attending to distant but structurally related crop features.

Model optimization. The proposed architecture still has room for further optimization to achieve better balance among segmentation performance, navigation line accuracy, and computational efficiency. Beyond traditional CNN-based approaches, several emerging techniques warrant exploration, including Transformer-based architectures that capture long-range dependencies (Xie et al., 2021a; Ravi et al., 2024), evolutionary neural architecture search methods with multipopulation mechanisms for automated design optimization (Yasmin et al., 2025), and recent Mamba-based models that offer efficient sequence modeling (Ruan et al., 2024). Investigating these advanced techniques could lead to architectures that better leverage the structural characteristics of crop rows while maintaining real-time performance on embedded platforms.

These directions aim not only to improve performance in challenging conditions but also to enhance the overall reliability and deployment readiness of vision-based navigation systems in real-world precision agriculture applications.

## 5 Conclusion

In this study, we proposed CCRDNet, a lightweight neural network designed to directly detect the central crop row as the navigation line in a human-like manner, thereby fundamentally simplifying the navigation process and enhancing robustness in complex and unseen agricultural environments. Unlike conventional multi-row extraction approaches that require feature clustering and post-processing, our method provides direct supervision on the navigation target itself, eliminating error propagation across multiple detection stages.

Trained on only 400 images (5.4% of the dataset) and tested on 7,367 images across eight crop species, CCRDNet achieved high line accuracy (95.57%), fast inference speed (86.76 FPS), and a minimal parameter count (0.033M). These results demonstrate strong generalization across different crops, growth stages, lighting conditions, and varying levels of weed interference, confirming the model’s capability to perform robustly in zero-shot environments that were never encountered during training.

Another key contribution of this work is the novel three-class annotation strategy: background, vegetation, and central crop row. By explicitly separating the general vegetation from the central crop row, this design provides the model with additional structural information that serves as an auxiliary supervisory signal to guide accurate localization. The consistent annotation width across all samples enables the model to learn the core structural characteristics of crop rows effectively, rather than being influenced by transient visual variations in foliage extent. Furthermore, this design alleviates class imbalance issues caused by the dominance of background pixels and prevents the model from overfitting to irrelevant background features.

Preliminary experiments using binary images as input also indicate that CCRDNet can be extended to handle dense, overlapping, or symmetric crop row arrangements, suggesting potential for broader applicability across various agricultural contexts. Overall, CCRDNet offers an accurate, efficient, and versatile solution for real-time crop row detection and autonomous navigation in precision agriculture, with navigation reliability depending on learned visual features rather than handcrafted rules.

## Data Availability

The datasets presented in this study can be found in online repositories. The names of the repository/repositories and accession number(s) can be found below: https://doi.org/10.5281/zenodo.15194034.

## Footnotes

1 Dataset is available at https://doi.org/10.5281/zenodo.15194034. Video results are also available at: https://1drv.ms/f/c/b0ba5174530596a7/EsuZvFgBt6BNvdKEfmYv5GEByPmfsXEFWzLDKPCjE7NDbA.
